# The History and Capabilities of Herbal Simples

**Published:** 1894-07-07

**Authors:** W. T. Fernie


### The History and Capabilities of Herbal Simples.

By W. T. Fernie, M.D.

### THE COMMON RAGWORT.

The Common Ragwort (Ragwurtz) is so called from being thought to possess aphrodisiac properties, just as the "pommes d'amour" are named "rage-apples." It is also known as staggerwort, perhaps because formerly employed to cure the staggers in horses. In some works the title is spelt staggwort; also it has been termed St. James's wort, because this saintly warrior was the patron of horses, and because it blooms about his day (July 25th). Furthermore, it has been designated stammerwort by certain writers, and seggrum. In early days it had a reputation for curing cancer, and was hence styled cankerwort. A poultice made of the fresh leaves was found singularly beneficial for relieving sciatica or hipgout, if ever so violent; and a decoction of the root was famous in former times for inward wounds and bruises. Gerarde said "It is good for green sores and old filthy ulcers which are not scoured, mundified, and made clean. In Ireland the plant is dedicated to the fairies, and is known with its golden-blossomed flower as the Fairies' Horse." In the Isle of Man it is used as a protection against infectious diseases, and by the Greeks of old it was believed to be potent against the spells of witches. In the neighbourhood of Liverpool persons now call this herb fleanart. Like its congener, the common groundsel, it has lancinated juicy leaves, which possess a bitter saline taste, and yield earthy potash salts abundantly.

